# Establishment of reference intervals of monomeric prolactin to identify macroprolactinemia in Chinese patients with increased total prolactin

**DOI:** 10.1186/s12902-021-00861-z

**Published:** 2021-10-07

**Authors:** Yao Hu, Jiajin Ni, Buyue Zhang, Wei Cheng, Huating Zhang, Hongying Ye, Lijin Ji, Bin Lu, Ming Guan

**Affiliations:** 1 Department of Laboratory Medicine, Shanghai Medical College, Huashan Hospital, Fudan University, 200040 Shanghai, China; 2 Department of Nursing, Shanghai Medical College, Huashan Hospital, Fudan University, 200040 Shanghai, China; 3 Department of Endocrinology, Huashan Hospital, Shanghai Medical College, Fudan University, 200040 Shanghai, China

**Keywords:** hyperprolactinemia, macroprolactinemia, reference interval

## Abstract

**Background:**

Macroprolactin is responsible for pseudohyperprolactinemia and is a common pitfall of the prolactin immunoassay. We aimed to determine the frequency of macroprolactinemia in Chinese hyperprolactinemic patients using monomeric prolactin discriminated by precipitation with polyethylene glycol (PEG).

**Methods:**

Post-PEG monomeric prolactin gender-specific reference intervals were established for the Elecsys immunoassay method (Roche Diagnostics) using sera from healthy female (*n* = 120) and male (*n* = 120) donors. The reference intervals were validated using 20 macroprolactinemic (as assessed by gel filtration chromatography (GFC)) sera samples, and presence of monomeric prolactin was discriminated by GFC. Patients with high total prolactin were then screened by PEG precipitation to analyze macroprolactin. The demographic and biochemical details of patients with true hyperprolactinemia and macroprolactinemia were compared.

**Results:**

Reference intervals for monomeric prolactin in females and males were 3.4–18.5 and 2.7–13.1 ng/mL, respectively. Among 1140 hyperprolactinemic patients, macroprolactinemia was identified in 261 (22.9 %) patients while the other 879 (77.1 %) patients were diagnosed with true hyperprolactinemia. Menstrual disturbances were the most common clinical feature in both groups. Galactorrhea, amenorrhea, and visual disturbances occurred more frequently in true hyperprolactinemic patients (*P* < 0.05).

**Conclusions:**

The prevalence of macroprolactin in Chinese patients with hyperprolactinemia was described for the first time. Monomeric prolactin concentration, along with a reference interval screening with PEG precipitation, provides a diagnostic approach for hyperprolactinemia with improved accuracy.

## Introduction

Heterogeneity has been described in the molecular size of prolactin in the majority of serum samples from healthy individuals and patients with hyperprolactinemia. Three major prolactin variants can be characterized, including monomeric, dimeric, and polymeric isoforms [1, 2]. Immunological and biological activity of prolactin in vivo may be almost exclusively attributed to the monomeric form [3]. Macroprolactin has a molecular weight > 100 kD and is a form of prolactin that is complexed with its autoantibody [4]. Macroprolactinemia is mostly considered an extra-pituitary phenomenon. Patients with macroprolactinemia experience mild and nonspecific symptoms of hyperprolactinemia [3]. In these patients, monomeric prolactin concentrations are within reference values and most macroprolactin is confined to the vascular system, where it is biologically inactive [5]. Macroprolactin poses a major problem due to its interference with prolactin assays. This commonly results in misdiagnosis and mismanagement of patients, as well as wasted healthcare resources and unnecessary concern from both patients and clinicians [6]. High concentrations of macroprolactin appears to result from reduced clearance of antigen-antibody complexes of monomeric prolactin and immunoglobulin G. These complexes interfere with the interaction between prolactin and the capture and detection antibodies involved in the sandwich reaction of prolactin immunoassays [7, 8]. Consequently, it is essential for clinical laboratories to establish screening methods to detect macroprolactin and the monomeric prolactin component in all hyperprolactinemic serum samples [9].

Macroprolactinemia is primarily defined by a circulating prolactin level that is made up of greater than 60 % macroprolactin [9]. The reference method for quantifying bioactive monomeric prolactin in sera is gel filtration chromatography (GFC). However, because this method is cumbersome and expensive, polyethylene glycol (PEG) serum precipitation is a simple, economical, and rapid method and has been widely used as a screening method to differentiate macroprolactinemia from true hyperprolactinemia [10–12]. Moreover, the laboratory priority should be to quantitatively determine the bioactive monomeric prolactin level, rather than simply to measure the percentage of macroprolactin present [13, 14].

Che Soh, NAA et al. reported the overall global prevalence of macroprolactinemia was 18.9 % among patients with hyperprolactinemia, and the prevalence in the South-East Asian region was 12.7 % [15]. However, there have been no previous reports pertaining to the reference interval of monomeric prolactin based on the Chinese population. Therefore, we have undertaken this cross-sectional investigation to screen for macroprolactin with higher accuracy.

## Materials and methods

### Reference intervals of monomeric prolactin establishment and validation

For the monomeric prolactin reference interval study, 240 blood samples (120 females, 120 males) from healthy donors between 18 and 60 years old were collected into serum gel tubes (Vacutainer 5.0 mL; BD). Females who were in hormone treatment for menopause or using estrogen-containing contraceptives were excluded. Samples were left to coagulate at room temperature for at least 30 min and were then centrifuged at 3000 × g for 10 min within 2 h. The serum was then divided into aliquots and stored at -30 °C until analysis.

### Hyperprolactinemia specimens

In total, 4520 patients 18 to 80 years of age were recruited from the Department of Endocrinology, Huashan Hospital of Fudan University, between May and December 2020. The study included 1140 serum specimens from the 4520 cases with total prolactin levels above normal range (Males>15.2ng/mL, females>23.3ng/mL; this reference interval was validated by and used in the Department of Laboratory Medicine of Huashan Hospital). All samples were stored frozen at -30 °C until assay-specific prolactin measurements were carried out.

### Gel filtration chromatography

The presence of monomeric prolactin was identified by gel filtration chromatography as described previously [3]. Macroprolactinemia was defined when the content of macroprolactin components accounted for more than 50 % of the total prolactin [11]. By GFC, we identified 20 macroprolactinemia samples (15 female, five male) from 95 patients whose sera indicated the presence of biochemical hyperprolactinemia before PEG-precipitation.

### PEG-precipitation

PEG-precipitation was performed by adding 200 µL of serum to an equal volume of 25 % (w/v) PEG 6000 dissolved in 0.9 % normal saline. After vortexing for 10 min at room temperature, specimens were centrifuged at 3000 × g for 10 min. The post-PEG monomeric prolactin level was determined by multiplying the prolactin concentration in the supernatant by two to correct for the dilution with PEG. Prolactin recovery was calculated by dividing the post-PEG monomeric prolactin concentration by the total prolactin result. If the post-PEG monomeric prolactin was more than 60 % of total prolactin, the specimen was considered negative for macroprolactin. To validate the established post-PEG reference intervals, we compared the classifications of macroprolactinemia obtained using our reference interval to the those obtained using GFC.

### Prolactin assay

All prolactin concentrations were measured using the Elecsys® Prolactin II assay from Roche Diagnostics (Cobas 8000; Mannheim, Germany). Prolactin immunoassays were calibrated against the World Health Organization (WHO) third international standard for prolactin (IS 84/500). The assay has an intra-assay imprecision (coefficient of variation; CV) of 5.8 % and 2.9 % at prolactin concentrations of 6.2 and 34.3 ng/mL, respectively. The inter-assay CVs in our experiments were 3.0 % and 6.4 %. All analyses were conducted using standardized laboratory procedures.

### Demographic and biochemical details collection

All patient charts were reviewed to record clinical signs and symptoms associated with hyperprolactinemia, including galactorrhea, menstrual disturbances, visual disturbances and the evidence of pituitary adenoma by imaging studies.

### Statistical analysis

Means and standard deviations (SD) were calculated for continuous variables. Serum prolactin concentrations were non-normally distributed and therefore were presented as medians (M) and quartiles (QR). Serum monomeric prolactin reference intervals were defined as 95 % confidence limits. Patients were stratified under macroprolactinemia groups and true hyperprolactinemia groups, according to monomeric prolactin reference range after PEG treatment. The Chi-square test was used for categorical variables. Differences were considered statistically significant at a P value of less than 0.05. Data were analyzed using the SPSS 13.0 (SPSS Inc., Chicago, IL, USA).

## Results

### Reference intervals of post-PEG serum monomeric prolactin

Data were collected from eligible participants, including 120 females (age 18–55 years, median 31 years) and 120 males (age 18–60 years, median 34 years). The demographic characteristics, total prolactin, and post-PEG monomeric prolactin at baseline are presented in Table 1. Female and male total and monomeric prolactin data were non-normally distributed according to histograms. The 95 % reference intervals for absolute levels of monomeric prolactin in females and males, respectively, ranged from 3.5 to 18.4 ng/mL and from 2.5 to 13.8 ng/mL. The reference values of total and post-PEG monomeric prolactin in male and female sera are illustrated in Table 2, along with manufacturer-provided reference intervals. There were no significant differences between the established total prolactin reference intervals and manufacturer range in either gender. In terms of post-PEG monomeric prolactin, we observed significant differences between females and males (*P* < 0.05).

**Table 1:** Descriptive statistics for total prolactin and post-PEG monomeric prolactin (ng/mL) in serum samples from healthy females (*n* = 120) and males (*n* = 120)

Characteristics	Minimum	Maximum	Mean	Median	SD	2.5th percentile	97.5th percentile
Total prolactin, ng/mL							
Females	4.0	29.1	12.2	10.4	5.5	4.5	25.6
Males	3.1	17.7	10.8	11.7	3.6	4.0	16.5
Post-PEG monomeric prolactin, ng/mL							
Females	3.0	20.2	9.4	8.0	4.2	3.5	18.4
Males	2.2	15.0	8.7	9.4	3.0	2.5	13.8

**Table 2:** Reference intervals (95 %) for total prolactin and post-PEG monomeric prolactin (ng/mL) in serum samples from females and males

	Established parametric range		Manufacturer’s range	
	Female	Male	Female	Male
Total prolactin, ng/mL	4.5–25.6	4.0-16.5	4.8–23.3	4.0-15.2
Post-PEG monomeric prolactin, ng/mL	3.5–18.4	2.5–13.8	N/A	N/A

N/A, not available

### Validation of the established monomeric prolactin reference intervals

Discriminated by GFC, 20 macroprolactinemia samples were analyzed and categorized by the monomeric prolactin reference intervals along with recovery cutoff method. We found that all macroprolactinemia sera (20/20) classified by the post-PEG monomeric prolactin reference intervals (Table 3) had initially elevated prolactin levels due to macroforms. Moreover, the monomeric reference interval method showed elevated discordant classification. A prolactin recovery cutoff of 60 % following PEG precipitation detected 70 % of specimens with macroPRL (defined as ≥ 50 % macroprolactin as determined by GFC). Decreasing the post-PEG PRL recovery cutoff to 40 % maintained only 80 % detection (Table 3).

**Table 3:** Discordant classification of macroprolactinemia as determined using the post-PEG reference interval and recovery methods

	Macroprolactinemia samples, N	Macroprolactinemia classification, N (%)
Reference interval method	20	20 (100)
Recovery method cutoff, %		
40	20	16 (80)
60	20	14 (70)

### Frequency of macroprolactin in hyperprolactinemia

**Fig. 1:**
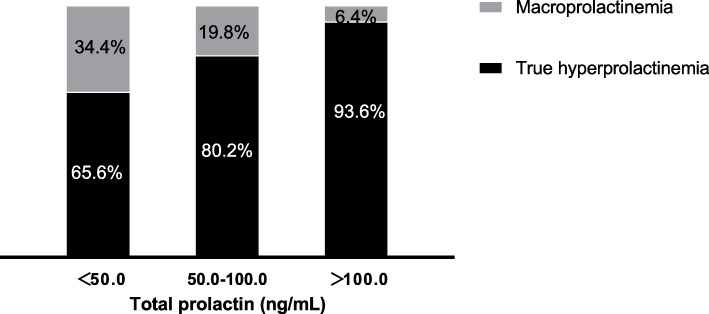
The prevalence of true hyperprolactinemia and macroprolactinemia among patients with increased total prolactin

Using PEG precipitation, 1140 patients with total prolactin levels above normal range were screened for macroprolactinemia. The median age of the patients was 33 years (interquartile range (IQR) = 27, 42) and the male/female ratio was 267/873 with female preponderance. The median total prolactin (ng/mL) was 29.1 (IQR = 21.3, 48.4) in males compared to 50.3 (IQR = 34.3, 88.7) in females. Macroprolactinemia was present in 22.9 % (*n* = 261) of hyperprolactinemic patients with normal monomeric prolactin concentrations. True hyperprolactinemia was identified in 77.1 % (*n* = 879) of patients. Based on serum total prolactin, all patients were divided into three groups. The prevalence of macroprolactinemia was 34.4 %, 19.8 %, and 6.4 % in groups with total prolactin below 50.0, 50.0-100.0, and above 100.0 ng/mL, respectively. The frequency of macroprolactinemia was the highest in patients with total prolactin less than 50.0 ng/mL. A significantly higher proportion of macroprolactinemia patients had total prolactin below 100 ng/mL (*P* < 0.001). A summary of the prevalence is presented in Fig. 1.

### Comparison of the demographic and biochemical characteristics of patients with true hyperprolactinemia and macroprolactinemia

The demographic and biochemical details of patients with true hyperprolactinemia and macroprolactinemia were compared (Table 4). Total prolactin was significantly higher (*p* < 0.001) in patients with true hyperprolactinemia compared to patients with macroprolactinemia, and this difference was maintained after treatment of serum with PEG.

**Table 4:** Comparison of demographic and biochemical characteristics of the patients

Characteristics	True hyperprolactinemia (*n* = 879)	Macroprolactinemia (*n* = 261)		P value
Age, years (IQR)	33(27, 43)	34(27, 42)	0.897	
Gender, N (%)				
Male	210(23.9 %)	38(21.8 %)	0.712	
Female	669(76.1 %)	204(78.2 %)	0.625	
Total prolactin, ng/mL (IQR)	50.7(33.1, 97.3)	32.9(26.3, 45.0)	< 0.001	
Monomeric prolactin, ng/mL (IQR)	37.2(24.5, 69.0)	12.0(8.1, 13.8)	< 0.001	
Imaging studies (MRI, CT) ^*^	201	30		
Adenoma detected, N (%)	120(59.7)	7(23.3)	< 0.001	
Microadenoma	54	5		

*MRI, magnetic resonance imaging; CT, computed tomography

**Fig. 2:**
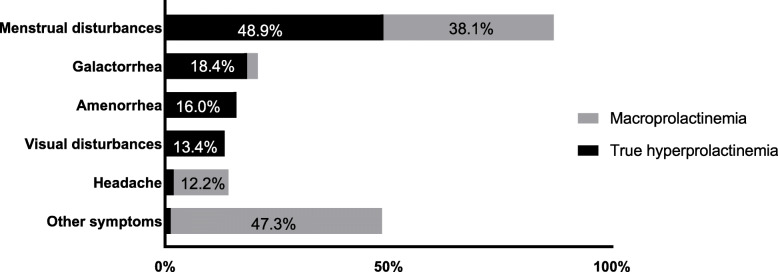
Clinical features of macroprolactinemic and true hyperprolactinemic patients

The clinical presentations were diverse and varied between macroprolactinemic and true hyperprolactinemic patients (Fig. 2). Menstrual disturbances were the main clinical feature in macroprolactinemic and true hyperprolactinemic female patients (38.1 % vs. 48.9 %). Galactorrhea was significantly more common in true hyperprolactinemic patients (*P* = 0.02), as well as amenorrhea (*P* = 0.01) and visual disturbances (*P* = 0.01). There was no significant difference in the incidence of headache between the true hyperprolactinemic and macroprolactinemic patients. However, in the macroprolactinemic group, the most common clinical symptoms were heat intolerance due to thyroidal illness, infertility, and obesity, in addition to less common symptoms of headache and galactorrhea.

Imaging studies [magnetic resonance imaging (MRI), computed tomography (CT)] were conducted in 201 true hyperprolactinemic patients. However, only 30 patients with macroprolactinemia were directed to further radiological examination. Medical data showed that 120 (59.7 %) of the 201 true hyperprolactinemic patients were confirmed to have pituitary adenomas. In contrast, in the group of macroprolactinemia cases, only five patients (17 %) were identified with pituitary microadenoma, as shown in Table 4.

## Discussion

In individuals with macroprolactinemia and normal levels of monomeric prolactin, nonspecific symptomatic progression was found during a 10-year clinical follow-up study. Therefore, macroprolactinemia can be considered a benign variant of hyperprolactinemia [16]. Failure to identify these individuals prior to or during laboratory prolactin assays leads to misdiagnosis, unnecessary investigation, inappropriate treatment, and ultimately an increased cost of management. Consequently, routine diagnostic macroprolactin screening in all hyperprolactinemic patients has been authoritatively recommended and financially justified due to reduced use of both imaging and dopamine agonist prescription in these patients [17].

It is difficult to differentiate between true hyperprolactinemia and macroprolactinemia based on clinical symptoms alone, as most of the patients with macroprolactinemia are also symptomatic. Without the use of PEG pretreatment, no commercial prolactin kits have been developed to accurately classify macroprolactin from true hyperprolactinemia. Only reporting with the recovery after PEG treatment lacks specificity, and may be inappropriate and misinterpreted in cases in which excess macroprolactin occurs simultaneously with supraphysiological concentrations of monomeric prolactin [18]. Screening for macroprolactinemia by reporting a monomeric prolactin concentration along with a PEG-specific monomeric reference interval is a recommended good practice for laboratories because the presence of excess monomeric prolactin is of clinical concern. This approach would enable more rigorous definition of macroprolactinemia than recovery cutoff method, and would prevent confusion when excess biologically active prolactin is present along with macroprolactin. Furthermore, this may be more valuable and easier to interpret than the reporting of macroprolactin along with a recovered prolactin concentration [18, 19].

Currently, 25 % PEG with molecular weight 6000 is commonly used in the precipitation of macroprolactin. Compared with PEG 8000, PEG 6000, with a relatively small degree of polymerization, can reduce the viscosity of the precipitant, increase the accuracy of sample loading and reduce damage to the analytical instrument [20]. In terms of selecting the PEG concentration, a previous study reported a significant constant bias between the 20 % and 25 % PEG macroprolactin precipitation methods [21]. Therefore, laboratories should establish the monomeric prolactin reference interval according to different concentrations of PEG 6000 carefully. This is an important clinical consideration if the presence of macroprolactin is based on absolute amounts of monomeric prolactin concentrations using post PEG reference interval.

In the present study, gender-specific monomeric prolactin reference intervals were established to avoid any confusion as to whether biologically active prolactin is also increased when excess macroprolactin is present. The application of a monomeric prolactin reference interval for the 25 % PEG 6000 precipitation procedure allowed for the detection of two cohorts of patients: one with true hyperprolactinemia and the other in whom hyperprolactinemia could be accounted for entirely by macroprolactin. True hyperprolactinemia is defined by the presence of excess monomeric prolactin in serum. In our study, macroprolactinemia was characterized by the presence of excess serum macroprolactin together with monomeric prolactin concentration in the normal reference interval. To our knowledge, this is the first study to compare unselected macroprolactinemic patients with true hyperprolactinemic patients among the Chinese population. A high incidence of macroprolactinemia in this study was identified to be 22.9 %. Other studies have reported very different macroprolactinemia incidences ranging from 10 to 45 % [22–24].

Our study demonstrated that, while menstrual disturbances occurred more frequently in patients with true hyperprolactinemia, they also occurred in 38.1 % of macroprolactinemic patients. Galactorrhea occurred less frequently in patients with macroprolactinemia compared to those with true hyperprolactinemia. Although this difference was statistically significant, it is clearly not sufficient to distinguish between the two groups. Out of a total of 30 macroprolactinemic patients who underwent MRI or CT scan, adenoma was detected in seven patients (23.3 %) and microadenoma was detected in five patients (17 %). Consistent with our observation, Hauache, OM et al. [19] observed that abnormal pituitary CT scans occurred in 21 % of macroprolactinemic patients. These results indicate that patients with macroprolactinemia can have microadenomas, and this should be evaluated with detailed clinical context and careful follow-up. And further based on our research results, the screening for macroprolactin should not be reserved for asymptomatic patients. Although this differs somewhat from the recommendations of the Endocrine Society Guidelines and guidelines published by the Spanish Society of Endocrinology and Nutrition and the Brazilian Society of Endocrinology and Metabolism [25–27], we insist that it is necessary to perform macroprolactinemia screening for those with an apparent idiopathic hyperprolactinemia, no obvious cause for the hyperprolactinemia, atypical clinical picture, conflicting prolactin results in distinct assays or delayed decline of serum prolactin levels with the usual doses of dopamine agonists.

Our study has certain advantages compared to previous studies. First, a large cohort of big data was used in the present study. Secondly, the reference interval of monomer prolactin based on Chinese population was established for the first time.

## Conclusions

It is important for asymptomatic hyperprolactinemia patients but also for those with an apparent idiopathic hyperprolactinemia and atypical clinical picture to perform routine screening for macroprolactinemia. The identification of macroprolactinemia by monomeric prolactin specific reference intervals based on pretreatment with PEG precipitation is an improved methodology with several advantages over the current standard methodology of GFC.

## Data Availability

All data generated or analyzed during this study are included in this published article and information about experimental sessions and results are available from the corresponding author on reasonable request.

## Abbreviations

PEG: polyethylene glycol
GFC: gel filtration chromatography
